# Energy management education for persons living with long COVID-related fatigue (EMERGE): protocol of a two-parallel arms target trial emulation study in a multicentre outpatient intervention setting with an online control group register

**DOI:** 10.1136/bmjopen-2024-098574

**Published:** 2025-02-07

**Authors:** Ruth Hersche, Andrea Weise, Emilia Riggi, Gian Luca Di Tanna, Marco Barbero

**Affiliations:** 1 Department of Business Economics, Health and Social Care, Rehabilitation Research Laboratory 2rLab, University of Applied Sciences and Arts of Southern Switzerland, Manno, Switzerland; 2 Department of Business Economics, Health and Social Care, University of Applied Sciences and Arts of Southern Switzerland, Manno, Switzerland

**Keywords:** Fatigue, REHABILITATION MEDICINE, Post-Acute COVID-19 Syndrome, Self-Management

## Abstract

**ABSTRACT:**

**Introduction:**

Energy management education (EME) is a manualised, evidence-based self-management education programme developed and delivered by occupational therapists for persons living with chronic disease-related fatigue. Studies have shown that EME can positively affect self-efficacy, fatigue impact and quality of life in persons with chronic conditions, while data on persons with long COVID are lacking.

The primary aim is to evaluate if adding EME to the standard care improves outcomes in persons with long COVID-related fatigue. The secondary aim is to explore the energy management behavioural strategies applied in daily routines and investigate the influencing factors of implementing behavioural changes. The third aim is to perform a cost-effectiveness analysis of EME.

**Methods and analysis:**

Using observational data, we will emulate a prospective two-parallel arms target trial to assess whether adding EME to the standard care is associated with improved outcomes in patients with long COVID-related fatigue. The estimated sample size to detect a post-intervention difference of 1.5 points in self-efficacy to implement energy conservation strategies with 90% power (0.05 alpha) is 122 people (1:1 ratio).

Persons with long COVID-related fatigue who follow EME as part of their standard care will be recruited and included in the experimental group (EG), while potential participants for the control group (CG) will be recruited from a register and prospectively matched to a participant in the EG by applying the propensity score technique. The ‘standard of care’ of the CG will include any intervention, except occupational therapy-based EME in peer groups. The causal contrast of interest will be the per-protocol effect. Four self-reported questionnaires (fatigue impact, self-efficacy in performing energy management strategies, competency in performing daily activities, health-related quality of life) will be administered at baseline (T0; week 0), after lesson 7 (T1; week 6), post-intervention (T2; week 14) and follow-up (T3, week 24). Our main assessment will be at T2. Disease-related and productivity cost data will be collected, and a cost-effectiveness profile of the EME intervention will be compared with standard care.

**Ethics and dissemination:**

Ethical approval has been obtained from the competent Swiss ethics commission.

Findings will be reported (1) to the study participants; (2) to patient organisations and hospitals supporting EMERGE; (3) to funding bodies; (4) to the national and international occupational therapy community and healthcare policy; (5) will be presented at local, national, and international conferences and (6) will be disseminated by peer-review publications.

**STRENGTHS AND LIMITATIONS OF THIS STUDY**

- This study will use a target trial emulation approach, which presents an alternative to a randomised controlled trial design when it is unethical or impractical.
- This study will deliver data on the effectiveness of an intervention recommended by guidelines and its cost-effectiveness.
- The problem of confounders in observational studies will be counteracted by using a propensity score matching technique, but not completely solved.

## Introduction

 Post-COVID-19 condition, also known as long COVID, occurs in individuals with a history of probable or confirmed SARS-CoV-2 infection, usually 3 months from the onset of COVID-19, with symptoms that last for at least 2 months, and cannot be explained by an alternative diagnosis.^1^ Prevalence estimates a range from 2.3% to 53% in adults.^2^ Long COVID may be more common in persons with a more severe acute illness or preexisting conditions.^3^

According to systematic reviews, fatigue, post-exertional malaise and dyspnoea are the most reported symptoms.^4 5^ Fatigue is experienced mainly as severe, impacting cognition, extended activities of daily living and social activities,^6^ which causes significant distress and disability to individuals, preventing their return to previous life routines, work and social life.^7^ Research on primarily mild-to-moderate and non-hospitalised SARS-CoV-2 cases reported that approximately 12%–23% of affected persons remained absent from work (or had long absence periods) at 3–7 months after acute disease.^2^

Currently, national and international guidelines, still mainly based on expert opinion, indicate a multidisciplinary approach for managing long COVID-related fatigue. This approach includes in addition to occupational therapy (OT), physiotherapy or psychological interventions, self-management education, peer support and symptom management strategies.^8^,^10^ The body of evidence supporting rehabilitation programmes for people with long COVID is gradually increasing, though it remains limited. According to a recent systematic review,^11^ which included studies up to May 2022, the evidence supporting physical training and breathing exercises to reduce fatigue and improve physical capacity and quality of life is limited. Another systematic review of registered interventional clinical trials for post-COVID syndrome, with a search date of September 2022, found that 168 out of 388 trials focused on physical rehabilitation, while only four trials focused on educational interventions.^12^ The most recent review and meta-analysis,^13^ which searched for randomised controlled trials (RCTs) until December 2023, included 24 trials with totally 3695 individuals. It identified “moderate certainty evidence that an online CBT programme probably improves fatigue and concentration, and a combined programme of physical and mental health rehabilitation probably increases the proportion of patients who experience recovery or important improvements” (p.9). Moderate certainty evidence was found for intermittent aerobic compared with continuous exercise, probably improving physical function. Despite OT, and particularly the use of energy management strategies, being one of the recommended interventions for persons with long COVID-related fatigue, data are missing on its effectiveness in managing the impact of fatigue on daily life and their costs.

Since 2021, many healthcare providers in Switzerland have started providing interprofessional in- and outpatient rehabilitation programmes for persons with long COVID. In a citizen science project in Switzerland in 2021, persons with long COVID discussed and identified the needs of affected persons. From a patient-centred perspective, rehabilitation and chronic care management, availability of interfaces for treatment continuity, availability of specific healthcare structures and knowledge among professionals were being identified as priorities for a research agenda and allocation of funding resources.^14^ Switzerland has not implemented a long COVID register yet.

Self-management education is the primary OT approach for persons with chronic fatigue syndrome^15^ and populations with chronic disease-related fatigue. Self-management refers to an individual’s ability to manage the symptoms, treatment, physical and psychosocial consequences and lifestyle changes inherent in living with a chronic condition.^16^

In 2017, group-based energy management education (EME)^17^ was developed for persons with multiple sclerosis-related fatigue. A detailed treatment manual and workbook for participants were written based on evidence-based intervention protocols for energy conservation^18^ and cognitive–behavioural therapy approaches,^19^ enriched with new elements from behavioural theories,^20 21^ principles for adult education and the inclusion of opinions from users and OT experts.

Since the beginning of the COVID-19 pandemic, occupational therapists (OTs) played a role in treating people with acute and post-acute COVID-19 and post-COVID-19 conditions in different settings (acute care, inpatient rehabilitation and outpatient reintegration). High-impact symptoms such as fatigue or post-exertional malaise affect the performance in daily activities, social participation and well-being. Some EME-trained OTs have started to include persons with post-COVID-19 condition-related fatigue in their EME groups or used the EME materials as part of individual therapy. In May 2021, nine OTs reported that the EME protocol was feasible and appropriate, although some optimisations for persons with long COVID-related fatigue were necessary.^22^ Since March 2021, the Clinic for Neurorehabilitation Basel (REHAB) has provided interprofessional consultation and day-hospital treatment programmes for persons with long COVID. EME is the standard OT intervention for persons with fatigue. In this clinical context, we performed a feasibility study in 2022 using a mixed-method design^23 24^ to improve the treatment materials and to prepare this study with promising results.

The purpose of this project is threefold. The primary aim is to evaluate if adding EME to the standard care improves outcomes for persons with long COVID-related fatigue. The secondary aim is to explore the energy management behavioural strategies applied in daily routines after participating in EME and investigate the influencing factors of implementing behavioural changes. The third aim is to perform a cost-effectiveness analysis of EME.

## Methods and analysis

### Project status

The recruitment at the three intervention group sites and for the control group (CG) register started in July 2024. The time between registration in the study and matching for the CG depends on the recruitment rate of the EME participants. The first matching of 15 participants of the experimental group with 15 participants out of the CG register was performed in December 2024. Study participation lasts for each study participant approximately 24 weeks; therefore, data collection was not finished with any participant before submission of this article. The recruitment will be finished during late summer/early autumn 2025, and data collection will be finished at approximately the end of 2025.

Data analysis will begin at the end of 2025 after the last participant has completed T3. The total duration of the research project is 42 months.

### Study design

The gold standard for investigating the efficacy of a treatment is an RCT, which guarantees high internal validity by minimising clinician and patient biases. In contrast, pragmatic trials prioritise external validity by studying interventions in the context of broad-based and normal clinical practice conditions.^25^ In rehabilitation research, pragmatic or real-world trials are increasingly being conducted.^26^ They aim to identify the most effective intervention for a patient-centred primary endpoint.^27^

Considering the above elements of the current context, we will emulate a prospective interventional study that draws causal inferences on the effectiveness of adding EME to standard care using a target trial emulation approach.^28 29^ Ethical approval has been obtained from the competent Swiss ethics commission (2024–00938; Rif. CE 4596).

### Interventions

*Standard care:* any interventions tailored to the specific needs and goals that aim to support individuals with long COVID on their way back to normality, such as breathing exercises, individual OT, relaxation techniques, mindfulness, cognitive–behavioural therapy, physical exercise training and consultations by different health professionals (eg, physicians, cardiologists, pulmonologists, psychologists, dietitians and social workers). All participants will follow the standard care personalised to their needs and preferences. The CG will receive the standard care only.

*Energy management education (EME), revised outpatient version 2023:* this updated version integrates the findings of the feasibility study performed in 2022.^23 24^ EME aims to increase self-management skills for managing the available amount of energy and achieving a satisfactory and meaningful daily routine despite fatigue. During EME, participants learn about the factors influencing their energy level and experiment with skills to manage their energy using behavioural strategies (eg, pace, plan and prioritise activities and optimise communication, environment and ergonomic behaviour). Subsequently, they identify and implement tailored behavioural modifications. To support the reflection on and use of energy-managing strategies and new behaviours, the leading OTs use behavioural change techniques.^21^ The outpatient EME protocol consists of seven group sessions (90 min) once a week plus an eighth session approximately 8 weeks after the seventh one. The first, seventh and eighth sessions can be done in individual sessions (45 min) alternatively. Sessions are led by EME-trained OTs. The experimental group will receive personalised standard care plus EME. The overview of the topics and further details are presented in table 1.

**Table 1:** Intervention: energy management education, revised outpatient version 2023

Structure	Delivery modality/duration		Topics	Applied behavioural change techniques
Lesson 1	Individual or group face-to-face/45 min or 90 min.	Self-training tasks	Energy account	Shaping knowledge, experience exchange and social support, feedback and monitoring, compared behaviour and outcomes, goals and action planning, antecedents, self-belief
Lesson2–6	Group face-to-face (3–7 participants)/90 min /once per week		Break management.Occupational balance.Successful communication.Simplifying.Think organised.	
Lesson 7	Individual or group face-to-face/ 45 or 90 min.		Apply in daily life	
Lesson 8 (Booster)	Individual or group, approx. 8 weeks after lesson 7		Review and reinforce	

At the University Hospital Bern (INSEL) and Clinic for Neurorehabilitation Basel (REHAB), the intervention starts with an individual lesson (45 min), whereas at University Hospital Zürich (USZ), the first lesson is already delivered in groups (90 min). With a frequency of one lesson per week, lessons 2–7 are provided (duration 90 min, appr. five participants). EME finishes with a group lesson, approximately 8 weeks after lesson 7. REHAB provides lessons 7 and 8 as individual lessons of 45 min. The EME-leading OTs use the treatment manual, which describes in detail the tasks of each lesson. All EME participants receive the workbook, which contains relevant information on the topics covered in each session, including worksheets and space for personal notes plus self-training tasks.

### Setting and recruiting

*Experimental group (EME, outpatient version 2023, plus standard care):* persons with long COVID-related fatigue following EME (revised edition 2023) in an outpatient setting at USZ, INSEL or REHAB, as part of their clinical routine and standard care, will be informed about this ongoing research and will be kindly asked if they are willing to participate in this project. If individuals agree, have signed the consent form and meet the inclusion/exclusion criteria checked by the study therapist on site, they will be included in the experimental group (EG). In these three public institutions, a group of trained OTs leads continuously EME groups and has received up-to-date training for the EME version 2023 in Spring 2024.

*CG (standard care*): a register of potential CG participants will be created through a contact with the Altea Network for Long COVID Association (ALTEA) and the patient organisation Long Covid Switzerland (LCS), both of which are partners in the study project and have been involved since the design phase. Using the contacts of ALTEA, LCS and specialised healthcare providers, information about the study will be disseminated, allowing interested persons to register themselves through a public link or QR code and to participate in a first online survey. Registration for the study has been open since 15 July 2024 and will remain open until summer 2025. Thereafter, based on the sociodemographic characteristics and clinical history of COVID-19, the CG participants will be selected from the register by their propensity score (PS) and matched with EG participants. Subsequently, the selected persons from the CG will be screened for eligibility, asked for their informed consent and included in the study.

### Eligibility criteria

The target study population consists of individuals aged 18 years or older, living in Switzerland, with a post-COVID-19 condition and a confirmed experience of fatigue. Eligible participants must not have major depression and/or significant cognitive impairments, must have sufficient ability to understand and speak one of the national languages and must provide signed informed consent. Persons are not eligible if they have previously participated in an EME group, have insufficient comprehension of one of the national languages or have a history of severe neurological diseases (eg, stroke, multiple sclerosis, Parkinson’s disease) or mental health disorders (eg, schizophrenia, major depression, or dementia).

### Objectives

The primary aim is to compare the effectiveness of the standard care for persons with long COVID-related fatigue plus participating in an EME group (revised edition 2023), with the standard care without EME at post-intervention (T2; week 14).

The secondary aim is to assess the energy management behavioural strategies applied in daily routines in persons living with long COVID, their feasibility and the factors influencing behavioural changes (T3, week 24).

The third aim is to evaluate the cost-effectiveness profile of the intervention by performing an economic evaluation alongside the trial.

### Outcomes

#### Primary outcomes

- The impact of fatigue on physical, mental and social activities of daily routines will be measured with the Modified Fatigue Impact Scale.^30^ It contains 21 items, on a scale from 0 (never) to 4 (almost always), thus total ranging from 0 to 84.
- Self-efficacy in performing energy management strategies is the most proximal outcome of EME. Increased perceived self-efficacy in performing energy management behaviours and strategies correlates to the impact of fatigue in daily life and predicts behavioural changes after patient education. This will be measured using the ‘Self-Efficacy for Performing Energy Conservation Strategies Assessment’.^31^ This self-report questionnaire reports the mean scores of perceived confidence in performing 14 different energy management strategies (1=low confidence, 10=high confidence). It is a valid and reliable instrument with a minimal clinically important difference of 0.92 in people with chronic conditions and fatigue.^32^

#### Secondary outcomes

- The Occupational Self-Assessment (OSA 2.2^33^; focuses on perceived competency to perform 21 daily tasks, which scores from 1 (I have many problems) to 4 (I do extremely well). The raw data are converted to interval levels (0–100). OSA has been validated in German and has shown the ability to detect changes in self-reported competence for daily activities over time.^34^
- Health-related quality of life will be measured by using the Short-Form 12 Item Health-Related Quality of Life Survey. This survey is a practical, reliable, valid and often used measure of physical and mental health. It assesses the same eight health domains as the SF-36,^35^ but with only one or two questions per domain: Physical Functioning, Role-Physical, Bodily Pain, General Health, Vitality, Social Functioning, Role-Emotional and Mental Health.^36^ Each health domain score contributes to the Physical Component Summary and Mental Component Summary scores.
- The Behavioural Changes in Energy Management Survey will be administered to describe the stable use of energy management strategies in daily life. This survey consists of eight questions and documents changes in using energy management strategies in daily routines, the effectiveness of stable strategy use (VAS 0–10), the simplicity of implementing them (VAS 0–10) and the actual stage of change. This survey has been used in two previous studies^23 37^ and was feasible (complete filled-out questionnaire response rate: 70%, average response duration: 10 min).

For the *cost-effectiveness* profile of the EME intervention assessed vs the standard care comparator, a self-reported questionnaire will be administered at several measure points to collect disease-related and productivity cost data allowing a more comprehensive assessment of the cost dimension. The dimensions for which data are collected include not only the ability to work and its remuneration and medical costs but also changes in costs, for example, mobility, leisure time and support at home.

### Patient and public involvement

With Altea Network for Long COVID Association (ALTEA) and the patient organisation Long Covid Switzerland (LCS) as project partners, the two relevant patient associations in Switzerland are part of this research project during all steps from the very beginning until the end. They participated during the protocol development like giving feedback about targeted research questions and outcome measures (eg, adequacy and burden), co-developed most patient information (informed consents, flyers, posts for social media, etc), are actively involved in the recruitment for the CG register and are giving feedback and meanings from the patient community about our study.

These two associations will be participating in the interpretation of the results of this study and their dissemination by co-authorship at scientific publications and congress participation and by having the lead in the dissemination to the patient community as to the public and politics.

Voluntary study participants’ feedback was and will be taken seriously and has resulted in minor changes. Study participants and participants in the CG register will be informed about the results of the study and their dissemination.

### Data collection

All questionnaires and surveys will be available in the three national languages (German, French and Italian) for both groups and at all time points online via Redcap (http://www.project-redcap.org/). If preferred, they will also be available on paper, either delivered personally by the local investigator or sent by post. Self-reported questionnaires documenting the outcomes of interest will be collected at different time points. In tables2  3, all procedures and data collection time points involving the CG and the EG are listed, respectively.

**Table 2:** Register and control group: timeline of contacts and assessments

Timeline contacts	Register	Study period control group (CG)						
	Enrolment		T0		T1		T2	T3
Weeks	>14	-2	0	4	6	9	14	24
Information about register and study procedures	x							
Informed consent for register	x							
Sociodemographic characteristics	x							
Clinical history of (post-)COVID-19-condition	x						x	x
Matching		x						
Check study inclusion/exclusion criteria		x						
Informed consent for CG participation		x						
Outcomes								
MFIS			x		x		x	x
SEPECSA			x		x		x	x
OSA			x		x		x	x
SF-12			x		x		x	x
BCEM-S								x
Disease-related costs				x		x	x	x

BCEM-SBehavioural Changes in Energy Management SurveyMFISModified Fatigue Impact ScaleOSAOccupational Self-AssessmentSEPECSASelf-Efficacy for Performing Energy Conservation Strategies AssessmentSF-12Short-Form 12 items

**Table 3:** Experimental group: timeline of contacts, assessments and energy management education intervention

Timeline contacts	Study period experimental group (eg,)							
	Enrolment		T0		T1		T2	T3
Weeks	>14	-2	0	4	6	9	14	24
Announced to EME at USZ, REHAB, INSEL		x						
Screening and inclusion into EME		x						
Written information about the study		x						
Check study inclusion/exclusion criteria			x					
Informed consent			x					
Sociodemographic characteristics			x					
Clinical history of (post-)COVID-19-condition			x				x	x
EME intervention (lessons 1–8)			L1-2-3-4-5-6-7-------8					
Outcomes								
MFIS			x		x		x	x
SEPECSA			x		x		x	x
OSA			x		x		x	x
SF-12			x		x		x	x
BCEM-S								x
Disease-related costs				x		x	x	x

BCEM-SBehavioural Changes in Energy Management SurveyEMEenergy management educationMFISModified Fatigue Impact ScaleOSAOccupational Self-AssessmentSEPECSASelf-Efficacy for Performing Energy Conservation Strategies AssessmentSF-12Short-Form 12 itemsUSZUniversity Hospital Zürich

Reasons for discontinuation are the following: (1) withdrawal of informed consent, (2) participants from the CG (without EME) starting in an OT-EME group during the data collection phase and (3) participants of the EG (with EME) missing more than three of the eight EME lessons. All data collected until withdrawal will be used in the analysis. If a participant decides to withdraw from the follow-up, the reasons for the withdrawal will be recorded for the subsequent analysis respectively the interpretation of the results. We will make all attempts to closely follow up participants and therefore minimise missingness throughout data collection. At each time point, participants of both groups will be invited to fill in the questionnaires via Redcap by presenting or e-mailing the correct web link and, if necessary, up to two reminders after 4–5 days each. No reaction will lead to a reminder for contacting the principal investigator, if they need any support to continue with the study, with a clear statement, that they are not obliged to answer this reminder or justify their drop-out if they do not want to.

### Data management

The local investigators at USZ, REHAB, INSEL and EME-OTs will be carefully trained by the principal investigator in their research-relevant tasks. All data collected on paper are entered into Redcap by the local investigator and regularly checked for accuracy by the principal investigator. Project data will be handled with the uttermost discretion and is only accessible to authorised personnel who require the data to fulfil their duties within the scope of the research project. On the case report forms and other project-specific documents, participants are only identified by a unique participant number. At the end of this project, we will remove any direct identifiers in the data before depositing the anonymised data on Zenodo Repository assigned with a persistent identifier (DOI).

### Statistical methods and analysis

#### Sample size

The target study population are people with post-COVID-19 condition living in Switzerland. To calculate the sample size of this study, we used data from two previous studies. In the feasibility study with persons with long COVID-related fatigue, the mean difference of the primary outcome (self-efficacy in performing energy conservation strategies) at T2 (post-intervention) was 1.9 (95% CI: 2.89 to 0.90).^23^ We hypothesised, therefore, a difference of 1.5 points at T2 and conservative assumption of a SD of 2.2 points.^38^ Including a follow-up rate of 24% at T2, the estimated sample size to detect a difference of 1.5 points with 90% power (0.05 alpha) is 122 persons. Therefore, we aim at a total of 122 persons included in the study (eg, 61 and CG: 61).

#### Statistical analysis

We will emulate a prospective interventional study that makes causal inferences on the effectiveness of adding EME to the standard care using a target trial emulation approach (see table 4).^28 29^

**Table 4:** Target trial protocol components

Protocol component	Emulated target trial
Eligibility criteria	Inclusion criteria: Living in Switzerland Age ≥18 years Diagnosed with post-COVID-19 condition Confirmed experience of living with fatigue (MFIS, cut-off >38)^47^ No major depression (Beck depression inventory (BDI)-FS cut-off <8)^48^ No major cognitive impairments (Mini-Mental State Examination (MMSE) cut-off of ≥26 for the experimental group and MMSE-telephone version for the control group (CG), cut-off≥21)^49 50^ Ability to understand and speak German, French or Italian and to fulfil the assessments Ability to give informed consent as documented by signature Exclusion criteria: Participation in an energy management education (EME) group or starting it during the study duration (only CG) History of severe neurological diseases (eg, stroke, MS and Morbus Parkinson’s disease) or mental health disorders (eg, schizophrenia, major depression, schizophrenia or dementia) Inability to follow the procedures of the study, for example, due to language problems, psychological disorders, dementia, etc.
Recruitment	Experimental group (eg,) participants at three study sites (INSEL, USZ, REHB) consecutive ongoing from mid-July 2024 up to the required number of 61 participants; maximum until end of 2025 A register for potential CG participants disseminated through the national patient organisations and specified healthcare providers
Follow-up	24 weeks after enrolment in the study
Outcomes	Impact of fatigue on physical, mental and social activities of daily routines measured by the Modified Fatigue Impact Scale Self-efficacy in performing energy management strategies measured using the Self-Efficacy for Performing Energy Conservation Strategies Assessment Perceived competency to perform 21 daily tasks measured by the Occupational Self-Assessment Health-related quality of life using the Short-Form 12 Item Health-Related Quality of Life Survey Description of the stable use of energy management strategies in daily life using the Behavioural Changes in Energy Management Survey Cost-effectiveness profile of the EME intervention plus the standard care vs standard care only: a self-reported questionnaire about disease-related and productivity costs
Interventions to be compared	Standard care: any interventions tailored to the personalised needs and goals of persons with long COVID Standard care plus occupatinal therapist-EME-group participation
Causal contrast	Per-protocol effect
Analysis plan	Each participant in the EG will be matched (1:1 ratio) to a participant in the CG by applying the optimal matching method using propensity score as a distance measure Outcomes will be analysed using an adjusted repeated measures mixed-effects linear regression model Cost-effectiveness by performing an economic evaluation

To emulate random assignment, each participant in the EG will be prospectively matched to a participant in the register according to their baseline sociodemographic characteristics (sex and age) and clinical history of long-COVID-related fatigue. We will choose 1:1 optimal matching using PS as a distance measure. We will employ a prospective matched design to compare outcomes between the experimental group and CGs. This ensures that CG participants are carefully selected in real time, concurrently with the enrolment of EG participants, to maximise group comparability and reduce confounding. For every 15 participants who are enrolled in the EG, we will prospectively match one CG participant to each EG participant. Optimal matching minimises the overall sum of pair-wise distances between treatment subjects and matched control subjects and is recommended when matching for the purpose of selecting well-matched controls for follow-up (eg, when the outcome values are not yet available).^39^

Descriptive statistics will be used for all outcome measures at each measurement time to summarise results. To ensure that PS matching adequately balances measured confounders, we will carefully assess and report standardised differences between treatment groups before and after matching.^40^ Standardised differences are widely used because they are unaffected by sample size and provide a consistent measure of imbalance. A standardised difference of less than 10% (0.1 in absolute value) will be considered indicative of a clinically relevant balance between groups. Variables with standardised differences of greater than 10% will be flagged for potential residual confounding and addressed in sensitivity analyses.

Our causal contrast of interest will be the per-protocol effect, so to analyse the effect of adding EME to the standard care on the dependent continuous variables for both primary and secondary outcomes, an adjusted repeated measures mixed-effects linear regression model will be used. In addition to the covariates already included in the matching phase, we are aiming to include additional potential covariates related to the clinical history of long-COVID (eg, fatigue intensity, fatigue impact) and socio-demographic data (eg, education, occupation status).

Using PS matching combined with regression methods is a robust approach to account for measured confounding. However, this approach does not address unmeasured confounding, which could bias the results. To acknowledge this limitation and mitigate its impact, we will perform a sensitivity analysis to evaluate the robustness of your findings to potential unmeasured confounding. We will perform a simulated bias analysis to estimate the potential impact of unmeasured confounding. We will specify plausible distributions for an unmeasured confounder and assess its potential effect on the treatment-outcome relationship.^41^ In addition, we plan to conduct an E-value analysis for assessing whether our results are likely robust even in the presence of unmeasured confounders.^42^ The E-value quantifies the minimum strength of association that an unmeasured confounder would need to have with both the treatment and the outcome (beyond measured confounders) to explain away the observed treatment effect.^43^

Cost-effectiveness of EME intervention against the standard care only will be evaluated by performing an economic evaluation, using the direct costs of delivering the intervention, disease-related and productivity as the cost input and various outcomes as health effects. A probabilistic sensitivity analysis will be performed to consider the uncertainty in the various input parameters and their effects on the decision-making process. After the maximal efforts have been employed to minimise the presence missing data, we will employ, beyond the default complete case analysis, a multiple imputation approach to assess the robustness of results.^44^ Statistical analysis will be performed using Stata (Stata, College Station, Texas, USA) and R (R Core Team (2023) R: A Language and Environment for Statistical Computing. R Foundation for Statistical Computing, Vienna, Austria; <https://www.R-project.org/>).

#### Missing data

After maximal efforts have been employed to minimise the presence of missing data, we will employ, beyond the default complete case analysis, a multiple imputation approach to assess the robustness of results.^44^

#### Volunteer bias

The fact that only persons who have actively registered themselves in the register can potentially get part of the CG will be carefully considered when interpreting and discussing the results of this study.

#### Selection bias

This common flaw of observational studies, we address by not using any information collected in the EG after the baseline period to determine which individuals from the register should be enrolled in the CG. By defining the recruitment of controls in two stages and having clear eligibility criteria, we create a structured approach to estimate causal effects even in the absence of true randomisation, based on an unbiased sample.

### Ethics and dissemination

Fatigue is a common and disabling condition for people with long COVID. The self-management education of persons enables them to implement new behavioural habits and engage more successfully in daily routines and social participation by managing their available energy. In the field of rehabilitation, this research will deliver urgently needed data on the effectiveness of an intervention recommended by guidelines for persons with long COVID and give a perspective on the potential and meaning of self-management education concerning health and well-being. Furthermore, the register of persons with a clinical history of long COVID can be used for further observational and evaluation studies, especially considering a long-term perspective. In the actual healthcare context in Switzerland, several factors prevent conducting an RCT or quasi-RCT aiming at investigating the effectiveness of EME in persons with long COVID-related fatigue: (1) in those institutions where the EME protocol with peer groups has been implemented as part of the standard care, it would not be feasible and/or ethical to introduce an alternative OT intervention acting as a CG intervention. (2) Enough patients must enter an OT service to continuously form and maintain EME groups. Randomisation into two groups would reduce the possibility of forming groups and prevent EME from being performed. (3) Furthermore, randomisation into a waiting list is not feasible because, in the case of EME, this would require at least 15 weeks, which would be an unnecessary additional patient burden.

Regarding OT, this research project will support the further development of evidence-based practice and use of standardised treatment protocols, which are until to date lowly applied in the daily practice of OTs in Switzerland.^45^ Data from the survey on the implemented energy management strategies will inform OTs about the most effective as well as the most difficult strategies to implement and provide insights into predicting factors and successful behavioural changes. This kind of knowledge in OT was, until now, missing or is, at present, only subjectively and empirically. Furthermore, OT researchers are asked to contribute their perspective to the development and discussion of the science of habit because a better understanding of the factors that support changes in habits and routines is also relevant in other health areas (eg, pain management, ergonomic behavior, stress management and lifestyle behaviour).^46^ The findings of this study will support the training of OTs in general and especially those delivering self-management education and support their skills in coaching and empowering patients and offering a more informed perspective for persons with long COVID.

This study will answer whether a higher effect justifies the effort of organising groups or if individual OTs are sufficiently empowering. At the same time, data from the cost-effective analysis will support rehabilitation managers, assurances and policymakers with data for informed decisions about group-based rehabilitation interventions.

The results of this study will be published as articles in scientific journals from various fields and presented at national and international congresses. In addition, we will disseminate the results through the social media channels of the partner organisations (Altea Network and Long-COVID-Switzerland), media dedicated to practitioners (OT and medical staff) and general media. In the training courses for EME-leading OTs and workshops for other healthcare professionals (eg, nurses, physicians), which AW and RH are organising in all parts of Switzerland and the neighboring countries, the results of this study will be integrated, and participants will function as multiplicators of the new knowledge.

## Data Availability

No data are available.
